# Chinese herbal Pulian ointment in treating psoriasis vulgaris of blood-heat syndrome: a multi-center, double-blind, randomized, placebo-controlled trial

**DOI:** 10.1186/s12906-017-1631-5

**Published:** 2017-05-15

**Authors:** Nuo Li, Wenbin Zhao, Jianmin Xing, Jianping Liu, Guangzhong Zhang, Yunbi Zhang, Yuanwen Li, Wali Liu, Fei Shi, Yanping Bai

**Affiliations:** 1 Department of Dermatology, China-Japan Friendship Hospital, Beijing, 100029 China; 2 Department of Dermatology, Beijing Pinggu Hospital, Beijing, 101200 China; 3 Beijing Sinocro Pharma Science Co., Ltd., Beijing, 100024 China; 4 Center for Evidence-Based Chinese Medicine, Beijing University of Chinese Medicine, Beijing, 100029 China; 5 Department of Dermatology, Beijing Hospital of Traditional Chinese Medicine, Beijing, 100010 China; 6 Department of Dermatology, Dongzhimen Hospital, Beijing, 100007 China; 7 Department of Dermatology, Dongfang Hospital, Beijing, 100078 China; 8 Department of Dermatology, Guang’anmen Hospital, Beijing, 100053 China; 9 Department of Dermatology, Air Force General Hospital, PLA, Beijing, 100036 China

**Keywords:** Pulian ointment, Chinese herbal medicine, Blood-heat syndrome, Psoriasis vulgaris, Efficacy, Randomized controlled trial

## Abstract

**Background:**

Traditional Chinese medicine (TCM) has a long history in the treatment of psoriasis vulgaris. We aimed to evaluate the clinical efficacy and safety of Chinese herbal Pulian ointment in treating psoriasis vulgaris of blood-heat syndrome.

**Methods:**

A multicenter, randomized, double-blind, placebo-controlled trial was conducted. Participants with psoriasis vulgaris of blood-heat syndrome were blinded and randomized to receive Pulian ointment or placebo ointment twice daily for 4 weeks, with follow-up 8 weeks after treatment. Psoriasis Area Severity Index (PASI) scores, severity of each symptom and area of skin lesion and quality of life were assessed at baseline, 2 weeks, and 4 weeks. Adverse events were recorded during the study. SAS 9.4 software and SPSS 17.0 software was applied for data analysis.

**Results:**

A total of 300 participants with psoriasis vulgaris of blood-heat syndrome were assessed for eligibility, and 294 were randomly assigned to the Pulian ointment and placebo group from six study centers. Full analysis set (FAS): after 4 weeks of treatment, there were significant differences between groups in PASI score and the separate score of skin lesion area, favoring Pulian ointment group (*P* < 0.05). However, no significant differences were observed in scores of scaling, erythema and induration/thickness (*P* > 0.05). Per protocol set (PPS): There was no statistically significant difference in PASI score and separate score of each symptom and area of skin lesion between two groups (*P* > 0.05). Quality of life measured by Hamilton Anxiety Rating Scale (HAMA) and 36-Item Short Form Health Survey (SF-36) improved after treatment in both groups, but there was no significant difference between the two groups (*P* > 0.05). After being followed up for 8 weeks, the total relapse rates of the Pulian Ointment group and placebo group were 5.88 and 8.45%, respectively, and the difference was not statistically significant between the two groups (*P* > 0.05). No adverse event was observed in both groups throughout the study.

**Conclusions:**

Pulian Ointment seems effective and well tolerated in improving the PASI score and separate score of skin lesion area for patients with psoriasis vulgaris of blood-heat syndrome. Further research could build on the current study to explore whether other preparation forms and greater intervention intensity are necessary for better therapeutic effects.

**Trial registration:**

Chictr.org.cn Identifier ChiCTR-TRC-12002054.

**Electronic supplementary material:**

The online version of this article (doi:10.1186/s12906-017-1631-5) contains supplementary material, which is available to authorized users.

## Background

Psoriasis is a common chronic immune-mediated inflammatory disease of skin and joints. Psoriasis vulgaris is the most common type of psoriasis. Estimates of the worldwide prevalence of psoriasis range from of 2 to3 % [1]. In China, the incidence of psoriasis had increased from 0.12% in 1984 to 0.72% in recent years [2]. Although psoriasis is not a life-threatening disease, it can severely affect participants’ quality of life (QoL).

Currently, there is no cure for psoriasis. Conventional western medications, such as methotrexate (MTX), acitretin A and ciclosporin, are limited for long-term use due to their limited efficacy and/or various potential toxic and side effects. The introduction of new biologic agents has increased the therapeutic options for the treatment of psoriasis, however, because of their high price, potential side-effects and unclear long-term efficacy and safety new biologic agents have not been widely used.

Traditional Chinese medicine has a long history of treating psoriasis based on specific syndromes, such as blood-heat syndrome. Pulian Ointment, an external therapy of Chinese herbal medicine, is a prescription developed by a famous traditional Chinese medicine practitioner, Bingnan Zhao. Pulian Ointment is used to clear excess heat and dampness, and relieve swelling and pain. Since the opening of China-Japan Friendship Hospital in 1984, Pulian Ointment has been used as a hospital prescription in the treatment of blood-heat syndrome psoriasis, and has been registered with the Beijing Food and Drug Administration (Preparation approval number 97 [123] 448). Our previous retrospective study showed that 3 weeks of treatment of psoriasis vulgaris by Pulian Ointment combined with oral Chinese medicine was superior to 5% boric acid ointment group in the total effective rate, and no adverse events were reported during the study [3]. Xu J. et al. [4] found that Qinbai Ointment (another name of Pulian Ointment) was more effective in relieving the skin lesion, and decreasing Psoriasis Area Severity Index (PASI) scores of participants with psoriasis, compared with white petroleum jelly group, and an animal experiment demonstrated that Qinbai Ointment could inhibit vaginal epithelial cell mitosis indices of mice and promote the formation of epithelial granular layer in the caudal scales of mice. Zhang J. et al. [5] found baicalin (main component of Pulian ointment) could inhibit proliferation of HaCaT keratinocytes and the mRNA and protein levels of IL-8 in vitro.

The objective of this study is to explore the clinical efficacy and safety of Pulian ointment in the treatment of participants with psoriasis vulgaris of blood-heat syndrome by external application.

## Methods

### Study design

This was a multicenter, randomized, double-blind, placebo-controlled study conducted in six centers in China. The six centers were as follows: China-Japan Friendship Hospital, Beijing Hospital of Traditional Chinese Medicine (TCM), Dongzhimen Hospital Affiliated to Beijing University of Chinese Medicine, Dongfang Hospital Affiliated to Beijing University of Chinese Medicine, Guang’anmen Hospital and Air Force General Hospital and Chinese People’s Liberation Army (PLA). Participants were outpatients or inpatients of Department of Dermatology. All personnel involved in this study undertook a training course including the study protocol, investigators’ responsibility and research monitoring, and relevant skills such as TCM syndrome differentiation. Manual for Investigators were also provided to each center.

### Study participants

#### Diagnostic criteria

Diagnosis of psoriasis vulgaris was based on the *Clinical Dermatology* (3rd Edition) edited by Bian Zhao et al. [6]: acute onset; at early stage, it is characterized by red inflammatory papules in the size of millet or mung bean, and would gradually enlarge or fuse into well-demarcated brownish red plaque, with peripheral inflammatory blush, superficially covered by multi-layer dry silver scale, and punctuate bleeding when peeled (Auspitz’s sign).

Diagnostic criteria of TCM blood-heat syndrome was based on the *Guiding Principles for Clinical Research on New Drug of Traditional Herbal Medicine* (1st Edition) [7]: primary symptoms include continuous appearance of new rash (mainly papule and maculopapule) with basal skin in bright red color, punctate bleeding when peeled, and Koebner phenomenon. Accompany symptoms include various levels of itching, vexation, thirst or dry mouth, constipation, yellow urine, red tongue with yellow coat, and rapid pulse. Participants who have all the primary symptoms and any two accompany symptoms can be diagnosed as psoriasis vulgaris of blood-heat syndrome.

#### Inclusion and exclusion criteria

Participants were included in the study if they were diagnosed as psoriasis vulgaris of blood-heat syndrome based on diagnostic criteria of psoriasis vulgaris and TCM blood-heat syndrome; aged between 18 and 65 years old; had skin lesion area < 30%; and willing to sign the informed consent form.

Participants were excluded if they had infection or trauma; during pregnancy or delivery; systematic or external local application of corticosteroids or immunosuppressant within 1 week before inclusion; severe heart, liver or kidney diseases; mental diseases; allergic history of general or local allergic responses to any Chinese herbal medicine or matrical ingredient of Pulian ointment or placebo used in this study.

### Randomization and blinding

Central block randomization method was applied in this study. Participants were randomly allocated into Pulian Ointment group or placebo group for 4 weeks. Follow-up period lasted 8 weeks after study completion. The randomization ratio was 1:1 in blocks of 2. Random numbers were generated by an independent third party (Center for Evidence-based Chinese Medicine, Beijing University of Chinese Medicine) using SAS PLAN statement for random sequence generation. Then randomization codes made by the third party were labeled to the internal and external packages of Pulian Ointment and placebo. The codes were stored by the principle investigator and the third party, so in the event of severe adverse reaction, the randomization code and group allocation of the participant could be identified. Participants were assigned to receive Pulian Onitment or placebo in sequential fashion. All subjects and investigators who were also the outcome assessors were blind to the randomization sequence and treatment allocation until the completion of data analysis.

### Interventions

#### Preparation and administration of Pulian ointment and placebo

Pulian ointment and placebo were produced and packaged by China-Japan Friendship Hospital.

The ingredients of Pulian ointment include *Huang Bai* (Phellodendron amurense) powder 50 g, *Huang Qin* (Scutellaria baicalensis) powder 50 g, and white petroleum jelly 400 g. Pulian ointment was a prescription derived from *Clinical Experiences of Bingnan Zhao*, and often used to treat the following diseases: impetigo, acute and sub-acute eczema, scalds and burns, herpes simplex, psoriasis and erythroderma. Each box of Pulian ointment was 20 g.

The preparation of Pulian ointment was as follows: powdered *Huang Bai* (Phellodendron amurense) and *Huang Qin* (Scutellaria baicalensis) purchased by the Department of Pharmacy of China-Japan Friendship Hospital were made into dusting powder, and screened through a 100–200 mesh sieve; white petroleum jelly was melted, filtered and cooled to a semisolid state; put the above dusting powders were added to the semisolid white petroleum jelly, stirred well, and continued to cool it until solidification. Quality of the Pulian ointment was tested and controlled by the Department of Pharmacy of China-Japan Friendship Hospital.

The Placebo ointment, 20 g per box, was prepared by same method of Pulian ointment. The matching placebo had similar smell and color with Pulian ointment because *Shen Qu* (medicated leaven) was added to it. The quality of placebo was also tested and controlled by Department of Pharmacy of China-Japan Friendship Hospital. A test to distinguish Pulian ointment and the placebo was successfully conducted involving 10 healthy people, and 70% of the people tested could not distinguish between Pulian ointment and the placebo ointment.

The Pulian ointment and placebo was applied externally, twice daily in the morning and at night after 12 h, continuously for 4 weeks. Specifically, the ointment was applied externally to all lesions on all areas of the body. The unit of dosage of the ointment was with the fingertip unit, which was the amount of ointment that fits on the space from the distal interphalangeal crease to the fingertip of the index finger of an adult. One fingertip unit of ointment was enough to be applied to an area of skin twice the size of the flat of one adult’s hand with the fingers together. The ointment was applied to an area 2 mm larger than the skin lesion area to ensure complete coverage. The method of application was to apply a sufficient amount in terms of ‘fingertip unit’, lightly pat the ointment on the lesion and spread it to form a thin ointment membrane over the skin lesion, then pat the areas for 1–2 min until the ointment was absorbed by the skin as much as possible.

During the study, any other TCM, western medicines or other therapies for psoriasis were forbidden to use. If participants had any concomitant diseases, the corresponding medications or therapies should be continued to use. The name, dosage, frequency and duration of the medications or other therapies were recorded on the medical records of participants.

### Outcome measures

All outcome measures were assessed and recorded at baseline, 2 weeks and 4 weeks. Follow-up assessments were scheduled at 4 weeks and 8 weeks after the end of treatment. Any adverse events were recorded throughout the study. (see Additional file 1: Table S1).

### Primary outcome measures

1. Psoriasis Area Severity Index (PASI) score: PASI is a quantitative rating score for measuring the severity of psoriatic lesions based on area coverage and plaque appearance.
2. Severity of each symptom (i.e. scaling, erythema, and induration/thickness) and area of skin lesion was separately scored by PASI to compare the severity changes of each symptom of skin lesion after treatment between the two groups.

### Secondary outcome measures

1. Quality of life (QoL): Hamilton Anxiety Rating Scale (HAMA) and 36-Item Short Form Health Survey (SF-36) were used for the evaluation of QoL.
2. Safety: any adverse events were recorded during the study. The indexes include vital signs, local skin responses, blood routine examination, urine routine examination, liver and kidney functions and other potential adverse reactions.

### Statistical analysis

All statistical procedures were performed with SAS 9.4 software and SPSS 17.0 software (SPSS Inc, Chicago, IL). Full analysis set (FAS) and Per protocol set (PPS) were applied for data analysis. FAS was defined as an analysis set as complete as possible including all randomized participants who had at least one time observation after randomization under Intention-to-treat (ITT) principles. If there was partially missing data, last-observation-carried-forward (LOCF) method was applied by replacing missing values with the last non-missing value. PPS was defined as a subset of the participants in the full analysis set who were more compliant with the protocol (defined as who completed 80–120% dosage of the treatment regimen; had no major protocol violations; and had some minimum number of measurements of the primary outcomes).

Safety was evaluated by Safety Set (SS), which included those who received at least one dose of the investigational treatment and had data of safety assessment. A two-tailed test was applied for all hypothesis tests. *P* ≤ 0.05 was set as statistical significance. A one-tailed test was applied for the superiority test of the primary outcome measures. The effect size estimate and 95% confidence interval (CI) were presented for the results.

Numerical (continuous) variables were analyzed by repeated measures analysis of variance (ANOVA), while categorical and ordinal variables by Cochran-Mantel-Haenszel test. The adherence of participants and incidence of adverse events were analyzed by chi-square test or Fisher’s exact test.

Considering the variation in the assessment of PASI among the six centers due to the assessment being done by different investigators, Mixed Effect Model was used in this study to analyze the PASI scores and single characteristic scores of skin lesion so as to evaluate the changes from baseline, with baseline PASI score and therapeutic efficacy as Fixed Effect and centers as Random Effect.

## Results

### Study population

**Fig. 1:**
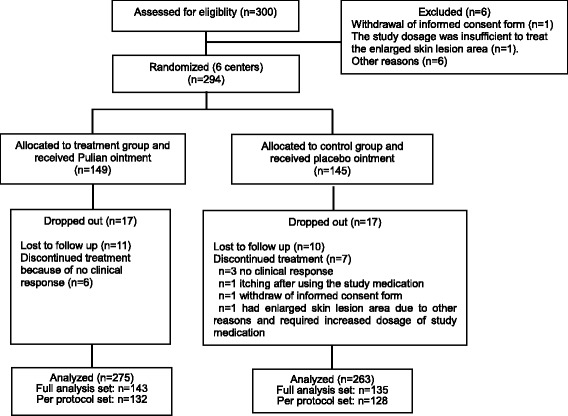
Flow chart of clinical trial

A total of 300 participants of psoriasis vulgaris of blood-heat syndrome were assessed for eligibility in this study from May 14th, 2010 to December 20th, 2011. After 6 participants were excluded due to not meeting the eligibility criteria, 294 participants were enrolled and randomized into Pulian ointment group (*n* = 149) or placebo group (*n* = 145). 34 participants dropped out the study, but 6 participants of Pulian ointment group and 7 participants of placebo group received treatment and completed one time point observation. Therefore, a total of 278 participants were in the ITT analysis, with 143 in the Pulian Ointment group and 135 in the placebo group; a total of 260 participants were included in per protocol analysis (PPS), with 132 in the Pulian ointment group and 128 in the placebo group. All participants who received at least one dose of Pulian Ointment or placebo were included in the safety analysis (SS) (Fig. 1).

At baseline, there was no statistically significant difference between the two groups in gender, systolic blood pressure (SBP), diastolic blood pressure (DBP), heart rate (HR), treatment history of psoriasis, skin lesion area, total SF-36 scores, HAMA scores, induration/thickness, area, erythema, scaling and PASI score (*P* > 0.05) (Table 1).

**Table 1:** Baseline characteristics of participants

	Pulian Ointment	Placebo
	(*n* = 143)	(*n* = 135)
Mean age, years (SD)	40 (13)	36 (12)
Male, n (%)	78 (54.55)	74 (54.81)
Had family history, n (%)	36 (25.17)	23 (17.16)
Mean HR (bpm) (SD)	74 (5)	74 (7)
Mean SBP, mmHg (SD)	118 (11)	119 (10)
Mean DBP, mmHg (SD)	78 (8)	77 (7)
Had treatment history of psoriasis, n (%)	83 (58.04)	78 (57.78)
Mean PASI score (SD)	6.4 (6.6)	6.8 (6.3)
Mean score of Area (SD)	1.2 (0.79)	1.26 (0.77)
Mean Erythema score (SD)	1.2 (0.63)	1.3 (0.66)
Mean Scaling score (SD)	1.17 (0.59)	1.29 (0.62)
Mean Induration/thickness score (SD)	1.37 (0.64)	1.5 (0.63)
Mean SF-36 score (SD)	78.39 (15.80)	80.25 (14.15)
Mean HAMA score (SD)	6.63 (6.55)	5.19 (5.29)

Abbreviations: *HR* heart rate, *bpm* beat per minute, *SBP* systolic blood pressure, *DBP* diastolic blood pressure, *PASI* Psoriasis Area Severity Index, *SF*-36, 36-Item Short Form Health Survey, *HAMA* Hamilton Anxiety Rating Scale, *SD* standard deviation

### PASI score and severity of each symptom including scaling, erythema, and induration/thickness and area of skin lesion

After 4 weeks of treatment, there were statistically significant differences in PASI score (FAS: MD − 0.70, 95% CI − 1.39 to − 0.02, *P* = 0.043) and area of skin lesion (FAS: MD 0.10, 95% CI − 0.19 to − 0.02, *P* = 0.02) between Pulian ointment and placebo group (*P* < 0.05), but there was no difference between the two groups in scaling, erythema and induration/thickness scores analyzed with FAS (*P* > 0.05). In PPS, there was no statistically significant difference in PASI score and severity of each symptom and area of skin lesion between the Pulian ointment and placebo group (*P* > 0.05) (Table 2).

**Table 2:** Changes from baseline in PASI score and severity of each symptom and area of skin lesion in patients with psoriasis of blood-heat syndrome receiving Pulian ointment or placebo

	Full analysis set (FAS)			Per protocol analysis (PPS)		
	(Mixed Effect Model with correction)			(Mixed Effect Model with correction)		
	Pulian Ointment (*n* = 143) (Mean change)	Placebo (*n* = 135) (Mean change)	Effect size estimate MD (95% CI; *P*-value)	Pulian Ointment (*n* = 132) (Mean change)	Placebo (*n* = 128) (Mean change)	Effect size estimate MD (95% CI; *P*-value)
PASI score						
2 weeks	−1.26	−0.98	−0.28 (−0.70, 0.14; 0.19)	−1.35	−0.99	−0.36 (−0.80, 0.07; 0.10)
4 weeks	−2.49	−1.78	−0.70 (−1.39, −0.02; 0.043*)	−2.51	−1.86	−0.65 (−1.31, 0.02; 0.056)
Area score						
2 weeks	−0.06	−0.03	−0.03 (−0.08, 0.01; 0.15)	−0.07	−0.03	−0.04 (−0.08, 0.01; 0.14)
4 weeks	−0.23	−0.13	−0.10 (−0.19, −0.02; 0.02*)	−0.17	−0.11	−0.06 (−0.14, 0.01; 0.09)
Scaling score						
2 weeks	−0.15	−0.15	−0.00 (−0.07, 0.06; 0.96)	−0.16	−0.15	−0.01 (−0.08, 0.06; 0.75)
4 weeks	−0.40	−0.32	−0.08 (−0.18, 0.03; 0.15)	−0.34	−0.29	−0.05 (−0.14, 0.05; 0.32)
Erythema score						
2 weeks	−0.19	−0.22	0.03 (−0.05, 0.11; 0.43)	−0.19	−0.21	0.02 (−0.05, 0.10; 0.54)
4 weeks	−0.44	−0.35	−0.08 (−0.19, 0.02; 0.12)	−0.39	−0.33	−0.06 (−0.16, 0.05; 0.29)
Induration/thickness score						
2 weeks	−0.19	−0.16	−0.02 (−0.11, 0.06; 0.59)	−0.20	−0.16	−0.04 (−0.12, 0.05; 0.39)
4 weeks	−0.42	−0.36	−0.06 (−0.18, 0.05; 0.29)	−0.35	−0.33	−0.02 (−0.12, 0.09; 0.72)

Note: *P < 0.05

### SF-36 and HAMA

After 4 weeks of treatment, the SF-36 scores increased within both the Pulian ointment and placebo groups compared to before treatment (*P* < 0.05), but the change scores were not statistically significant between the two groups both in FAS and PPS (*P* > 0.05). (Additional file 2: Table S2).

After 4 weeks of treatment, the HAMA scores decreased within both the Pulian ointment and placebo groups compared to before treatment (*P* < 0.05), but the change scores were not statistically significant between the two groups both in FAS and PPS (*P* > 0.05). (Additional file 2: Table S3).

### Safety

No adverse events were observed during the study. There was no significant difference in blood, urine, and liver and kidney function tests between the Pulian ointment and placebo groups.

### Adherence

The adherence rate was 84 and 87.3% in the Pulian Ointment and placebo group, respectively. There was no statistically significant difference between the two groups in adherence rate (*P* = 0.41).

### Follow up visit

Follow-up visits were at 4 weeks and 8 weeks after the end of treatment. 72 participants in Pulian Ointment group were willing to be followed up, of which 68 (94.44%) completed the follow up visits. 73 participants in placebo group were willing to be followed up, of which 71 (97.26%) completed the follow up visits.

In Pulian Ointment group, 5.88% (4/68) of participants who completed the follow up visits relapsed within 8 weeks, whereas in placebo group, 8.45% (6/71) of participants who completed the follow up visits relapsed within 8 weeks. There was no statistically significant difference between two groups in the relapse rate (*P* > 0.05).

## Discussion

This multi-center, double-blind, randomized, placebo-controlled trial found that Pulian ointment could significantly decrease the PASI score and the separate score of skin lesion area in patients with psoriasis vulgaris of blood-heat syndrome compared to the placebo after 4 weeks of treatment (FAS set). However, no statistically significant difference was found between the two groups in PPS set. The underlying reasons might be as follows. Firstly, psoriasis vulgaris of blood-heat syndrome is characterized by the aggravation stage of psoriasis and the lesions are less stable, so there were natural variations in psoriasis among participants who are at different stages of exacerbation or remission during the treatment course of 4 weeks in this study. Secondly, the matrix of both the Pulian ointment and placebo ointment was white petroleum jelly, accounting 80% of the ointment. Previous studies showed that white petroleum jelly has some therapeutic effects on psoriasis [8], which might reduce the differences in outcomes between the two groups. Thirdly, there was variation in measurement and the number of different investigators among different study centers. All these above factors might make it difficult to detect an effect in a randomized controlled trial.

Quality of life after 4-week treatment in this study was improved within both groups measured by SF-36 and HAMA, however, Pulian ointment group was not superior to placebo group in the improvement of quality of life. This could be related to the placebo effect, short duration, and subjective measurement (questionnaires).

During the 4 weeks of treatment in this study, no adverse events were observed in the Pulian ointment and placebo group, indicating the short-term safety of Pulian ointment in treating psoriasis vulgaris of blood-heat syndrome.

The potential mechanism of Pulian ointment in treating psoriasis vulgaris remains unclear. The ingredients of Pulian ointment include *Huang Bai* (Phellodendron amurense), *Huang Qin* (Scutellaria baicalensis) and white petroleum jelly. Pharmacological studies demonstrated that *Huang Bai* (Phellodendron amurense) contains alkaloids (berberine, tetrahydroberberine, magnoflorine, phellodendrine, jatrorrhizine, 4-tetrahydro jatrorrhizine, N-methyl hordenine, etc.), sterols (campesterol, 7-dehydro stigmasterol, β-sitosterol, etc.) and limonins (obacuonic acid, obacunone, obaculactone, etc.), which could inhibit proliferation and have antibacterial, anti-inflammatory and anticancer effects [9]. Choi YY et al. [10] found that Cortex Phellodendri amurensis could down-regulate nitric oxide (NO) and inducible nitric oxide synthase (iNOS) in vivo and in vitro, suggesting its potential application for attenuating inflammation-related diseases. Pharmacological studies demonstrated that *Huang Qin* (Scutellaria baicalensis) had anti-inflammatory, antioxidant, anticancer and immune-regulatory functions [11]. Currently, it is believed that the mechanism of *Huang Qin* (Scutellaria baicalensis) and its extract Baicalin in the treatment of psoriasis was the inhibition of the keratogenesis and cell proliferation [12], the activation of Jurkat T cells in-vitro [13] and the iNOS protein expression in fibroblast cells stimulated by cytokines, and reduce the generation of NO [14]. Yang WH et al. [15] found that Baicalin attenuated lipopolysaccharide induced inflammation and apoptosis of cow mammary epithelial cells by regulating NF-κB and HSP72. Wu J et al. [16] revealed that Baicalin cream possessed anti-inflammatory action in 2,4-dinitrofluorobenzene (DNFB)-induced contact hypersensitivity (CHS) mice, as well as keratinocyte differentiation-modulating activity in mouse tail test.

### Limitations

There were some limitations of this study. Though all personnel had training courses and Manual for Investigators of this study were provided to the six centers, there were variations among different centers in recruitment, adherence and measurement. The recruitment in some study centers was more difficult than that of others. The possible reason might be that people are hesitated to use Pulian ointment due to the yellow ointment stains. Though the adherence rate of Pulian ointment group (84%) was acceptable, we recommend future research apply some methods to prevent staining of clothes, such as covering the area with a gauze dressing. In addition, the outcome assessor was not the same investigator in each study center, resulting in variations of measurement of PASI.

Another issue is that 4-week treatment was too short to provide an assessment of the long-term efficacy and safety of Pulian ointment. Since psoriasis is a chronic, relapsing skin disease, the long-term effects of a medication will be an important concern of the patients with psoriasis. Future studies could consider longer treatment durations and follow-up periods.

Furthermore, it is unclear from this study that whether other preparation form (such as a cream matrix or other formulation) or greater intervention intensity was necessary.

## Conclusion

Pulian ointment is potentially safe and effective in improving the PASI score and the separate score of skin lesion area for patients with psoriasis vulgaris of blood-heat syndrome. Further research could build on the current study by more closely exploring the preparation forms. Investigation of a possible dose–response relationship between Pulian ointment and PASI scores is also warranted in the future.

## Additional files

Additional file 1: Table S1. Assessment procedures and time-points. (DOCX 14 kb)

Additional file 2: Table S2. Changes of SF-36 scores from baseline in Pulian ointment and placebo group. **Table S3.** Changes of HAMA scores from baseline in the Pulian ointment and placebo group. (DOCX 17 kb)

## Abbreviations

ANOVA: Analysis of variance
CI: Confidence interval
FAS: Full analysis set
HAMA: Hamilton Anxiety Rating Scale
HR: Heart rate
iNOS: Inducible nitric oxide synthase
ITT: Intention-to-treat
LOCF: Last-observation-carried-forward
MTX: Methotrexate
NO: Nitric oxide
PASI: Psoriasis Area Severity Index
PLA: Chinese People’s Liberation Army
PPS: Per protocol set
QoL: Quality of life
SBP: Systolic blood pressure
DBP: Diastolic blood pressure
SF-36: 36-Item Short Form Health Survey
SS: Safety set
TCM: Traditional Chinese medicine
