# Supplementary material for: Chinese herbal Pulian ointment in treating psoriasis vulgaris of blood-heat syndrome: a multi-center, double-blind, randomized, placebo-controlled trial
Source: BMC Complement Altern Med. 2017 May 15;17:264. doi: 10.1186/s12906-017-1631-5 (PMC5432985; doi:10.1186/s12906-017-1631-5)
Supplement: Supplementary file 1 — Assessment procedures and time-points. (DOCX 14 kb) [file 12906_2017_1631_MOESM1_ESM.docx]

**Additional file 1: Table S1.** Assessment procedures and time-points

| **Time-point** | **Week 0** | **Week 2** | **Week 4** | **Week 8** | **Week 12** |
| --- | --- | --- | --- | --- | --- |
| **Assessment** | Baseline assessment | Mid treatment assessment | End of treatment assessment | Follow-up assessment | Follow-up assessment |
| **Outcome measure** |  |  |  |  |  |
| Baseline characteristics | X |  |  |  |  |
| HR | X |  | X |  |  |
| SBP | X |  | X |  |  |
| DBP | X |  | X |  |  |
| PASI | X | X | X | X | X |
| SF-36 | X | X | X |  | X |
| HAMA | X | X | X |  | X |
| *Safety | X |  | X |  |  |
| Relapse rate |  |  |  |  | X |

Note: HR, heart rate; SBP, systolic blood pressure; DBP, diastolic blood pressure; PASI, Psoriasis Area Severity Index; SF-36, 36-Item Short Form Health Survey; HAMA, Hamilton Anxiety Rating Scale. *Any adverse events were recorded during the study. The indexed include vital signs, local skin responses, and other potential adverse reactions. Blood routine examination, urine routine examination, liver and kidney functions were tested before and after the treatment.
