# Supplementary material for: Chinese herbal Pulian ointment in treating psoriasis vulgaris of blood-heat syndrome: a multi-center, double-blind, randomized, placebo-controlled trial
Source: BMC Complement Altern Med. 2017 May 15;17:264. doi: 10.1186/s12906-017-1631-5 (PMC5432985; doi:10.1186/s12906-017-1631-5)
Supplement: Supplementary file 2 — Changes of SF-36 scores from baseline in Pulian ointment and placebo group. Table S3. Changes of HAMA scores from baseline in the Pulian ointment and placebo group. (DOCX 17 kb) [file 12906_2017_1631_MOESM2_ESM.docx]

**Additional file 2**

**Table S2. Changes of SF-36 scores from baseline in Pulian ointment and placebo group**

|  | Group | Baseline  Mean (SD) | Week 4  Mean (SD) | F (*P*-value) | | |  |  |
| --- | --- | --- | --- | --- | --- | --- | --- | --- |
|  |  |  |  | Comparison in group at baseline | Comparison in group at week 4 | Comparison between A and B at week 4 |  |  |
| FAS | A | 78.3892±16.15081 | 81.0676±14.93240 | 17.246 (0.000) | 0.740 (0.390) | 0.759 (0.385) |  |  |
|  | B | 80.3527±14.00227 | 82.1119±13.47347 |  |  |  |  | |
| PPS | A | 78.2629±16.14628 | 81.0789±14.98871 | 18.565 (0.000) | 0.868 (0.353) | 0.803 (0.371) |  |  |
|  | B | 80.3318±14.12718 | 82.1467±13.60031 |  |  |  |  |  |

Abbreviation: FAS: Full analysis set; PPS: Per protocol set; Group A: Pulian ointment; Group B: Placebo; SD; standard deviation.

**Table S3 Changes of HAMA scores from baseline in the Pulian ointment and placebo group**

|  | Group | Baseline  Mean (SD) | Week 4  Mean (SD) | F (*P*-value) | | |
| --- | --- | --- | --- | --- | --- | --- |
|  |  |  |  | Comparison in group at baseline | Comparison in group at week 4 | Comparison between A and B at week 4 |
| FAS | A | 6.2857±6.43802 | 5.4662±6.03217 | 10.794 (0.001) | 0.000 (0.996) | 3.530 (0.061) |
|  | B | 5.0611±5.20614 | 4.2443±4.80879 |  |  |  |
| PPS | A | 6.3333±6.43899 | 5.5076±6.03615 | 10.800 (0.001) | 0.000 (0.984) | 3.362 (0.068) |
|  | B | 5.1328±5.22768 | 4.2969±4.83322 |  |  |  |

Abbreviation: FAS: Full analysis set; PPS: Per protocol set; Group A: Pulian ointment; Group B: Placebo; SD; standard deviation.
